# Supplementary material for: UVR2 ensures transgenerational genome stability under simulated natural UV-B in Arabidopsis thaliana
Source: Nat Commun. 2016 Dec 1;7:13522. doi: 10.1038/ncomms13522 (PMC5146273; doi:10.1038/ncomms13522)
Supplement: Supplementary Information — Supplementary Figures 1-7 and Supplementary Tables 1 and 2 [file ncomms13522-s1.pdf]

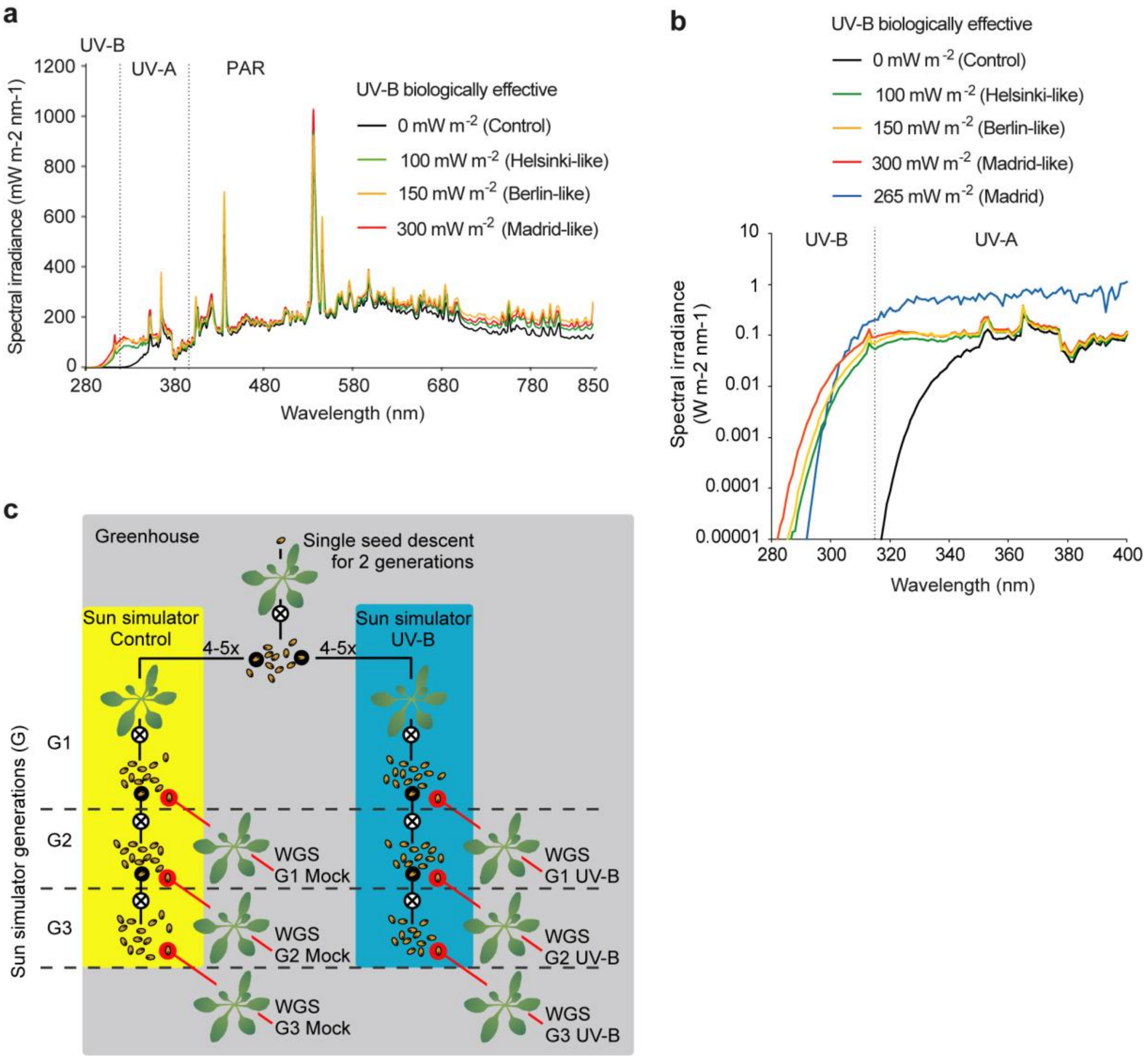

Supplementary Figure 1: Irradiation spectra and plant material genealogy.

(**a**) Spectral irradiance in sun simulators at 0, 100, 150, and 300 mW m^{-2} UV-B$_{BE}$. UV-B, UV-A and photosyntetically active radiation (PAR) spectra are divided by dotted vertical lines. (**b**) Spectral irradiance of the four UV-B regimes in sun simulators at UV-B and UV-A spectra. (**c**) Plant material genealogy. Seeds of each genotype were amplified by a single seed descent for two generations. From a genotype-specific common pool of seeds, 15 plants per treatment were grown until seed set (G1). From the pool of sun simulator generation 1 (G1) seeds, a single plant was propagated under the same conditions in G2 and G3. For genome analysis, a single progeny plant per originally irradiated plant was grown under greenhouse conditions without UV-B and whole genome sequenced (WGS). The overview of sequenced genomes, generations and treatments can be found in Table S1.

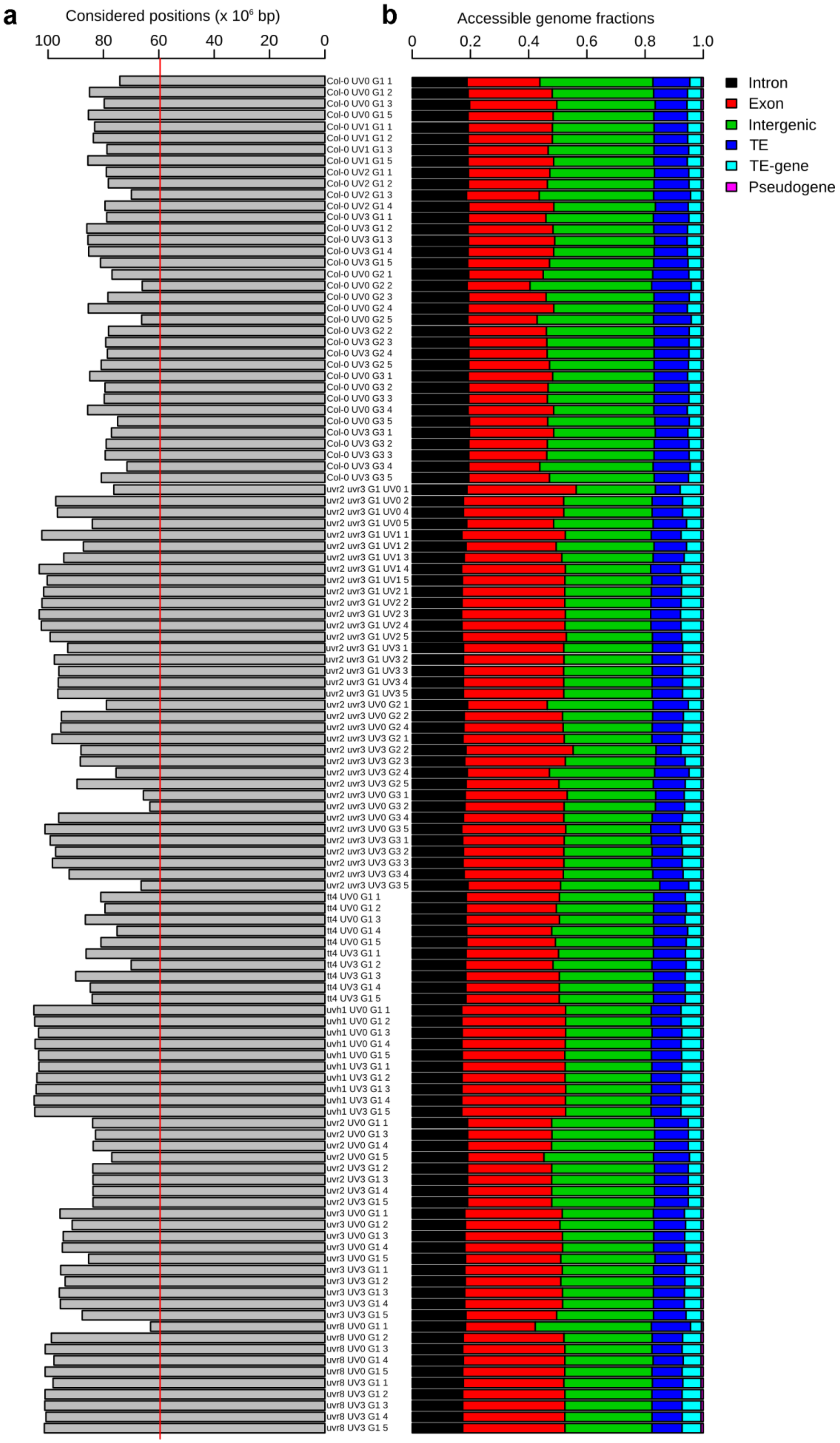

a
Considered positions (x 10⁶ bp)
100 80 60 40 20 0
b
Accessible genome fractions
0 0.2 0.4 0.6 0.8 1.0
Intron
Exon
Intergenic
TE
TE-gene
Pseudogene
Col-0 UV0 G1 1
Col-0 UV0 G1 2
Col-0 UV0 G1 3
Col-0 UV0 G1 5
Col-0 UV1 G1 1
Col-0 UV1 G1 2
Col-0 UV1 G1 3
Col-0 UV1 G1 5
Col-0 UV2 G1 1
Col-0 UV2 G1 2
Col-0 UV2 G1 3
Col-0 UV2 G1 4
Col-0 UV3 G1 1
Col-0 UV3 G1 2
Col-0 UV3 G1 3
Col-0 UV3 G1 4
Col-0 UV3 G1 5
Col-0 UV0 G2 1
Col-0 UV0 G2 2
Col-0 UV0 G2 3
Col-0 UV0 G2 4
Col-0 UV0 G2 5
Col-0 UV3 G2 2
Col-0 UV3 G2 3
Col-0 UV3 G2 4
Col-0 UV3 G2 5
Col-0 UV0 G3 1
Col-0 UV0 G3 2
Col-0 UV0 G3 3
Col-0 UV0 G3 4
Col-0 UV0 G3 5
Col-0 UV3 G3 1
Col-0 UV3 G3 2
Col-0 UV3 G3 3
Col-0 UV3 G3 4
Col-0 UV3 G3 5
uvr2 uvr3 G1 UV0 1
uvr2 uvr3 G1 UV0 2
uvr2 uvr3 G1 UV0 4
uvr2 uvr3 G1 UV0 5
uvr2 uvr3 G1 UV1 1
uvr2 uvr3 G1 UV1 2
uvr2 uvr3 G1 UV1 3
uvr2 uvr3 G1 UV1 4
uvr2 uvr3 G1 UV1 5
uvr2 uvr3 G1 UV2 1
uvr2 uvr3 G1 UV2 2
uvr2 uvr3 G1 UV2 3
uvr2 uvr3 G1 UV2 4
uvr2 uvr3 G1 UV2 5
uvr2 uvr3 G1 UV3 1
uvr2 uvr3 G1 UV3 2
uvr2 uvr3 G1 UV3 3
uvr2 uvr3 G1 UV3 4
uvr2 uvr3 G1 UV3 5
uvr2 uvr3 UV0 G2 1
uvr2 uvr3 UV0 G2 2
uvr2 uvr3 UV0 G2 4
uvr2 uvr3 UV3 G2 1
uvr2 uvr3 UV3 G2 2
uvr2 uvr3 UV3 G2 3
uvr2 uvr3 UV3 G2 4
uvr2 uvr3 UV3 G2 5
uvr2 uvr3 UV0 G3 1
uvr2 uvr3 UV0 G3 2
uvr2 uvr3 UV0 G3 4
uvr2 uvr3 UV0 G3 5
uvr2 uvr3 UV3 G3 1
uvr2 uvr3 UV3 G3 2
uvr2 uvr3 UV3 G3 3
uvr2 uvr3 UV3 G3 4
uvr2 uvr3 UV3 G3 5
tt4 UV0 G1 1
tt4 UV0 G1 2
tt4 UV0 G1 3
tt4 UV0 G1 4
tt4 UV0 G1 5
tt4 UV3 G1 1
tt4 UV3 G1 2
tt4 UV3 G1 3
tt4 UV3 G1 4
tt4 UV3 G1 5
uvh1 UV0 G1 1
uvh1 UV0 G1 2
uvh1 UV0 G1 3
uvh1 UV0 G1 4
uvh1 UV0 G1 5
uvh1 UV3 G1 1
uvh1 UV3 G1 2
uvh1 UV3 G1 3
uvh1 UV3 G1 4
uvh1 UV3 G1 5
uvr2 UV0 G1 1
uvr2 UV0 G1 3
uvr2 UV0 G1 4
uvr2 UV0 G1 5
uvr2 UV3 G1 2
uvr2 UV3 G1 3
uvr2 UV3 G1 4
uvr2 UV3 G1 5
uvr3 UV0 G1 1
uvr3 UV0 G1 2
uvr3 UV0 G1 3
uvr3 UV0 G1 4
uvr3 UV0 G1 5
uvr3 UV3 G1 1
uvr3 UV3 G1 2
uvr3 UV3 G1 3
uvr3 UV3 G1 4
uvr3 UV3 G1 5
uvr8 UV0 G1 1
uvr8 UV0 G1 2
uvr8 UV0 G1 3
uvr8 UV0 G1 4
uvr8 UV0 G1 5
uvr8 UV3 G1 1
uvr8 UV3 G1 2
uvr8 UV3 G1 3
uvr8 UV3 G1 4
uvr8 UV3 G1 5

Supplementary Figure 2: Genome accessibility.

(**a**) Number of base pair positions covered by ≥20 sequencing reads in individual sequenced genotypes. Genome was considered as suitable for analysis when at least 50% (vertical red line) of the assembled Arabidopsis genome (TAIR10; 119.1 Mbp) had this coverage. 10 genomes with lower coverage (see **Supplementary Table 1**) were excluded from analysis. (**b**) Proportions of accessible genome fractions within the considered positions for individual genomes. UV0, UV1, UV2 and UV3 corresponds to 0, 100, 150 and 300 mW m^{-2} UV-B_{BE}, respectively and G1, G2 and G3 to sun simulator generation. The last number is the genome replicate. Detailed information is provided in **Supplementary Table 1**.

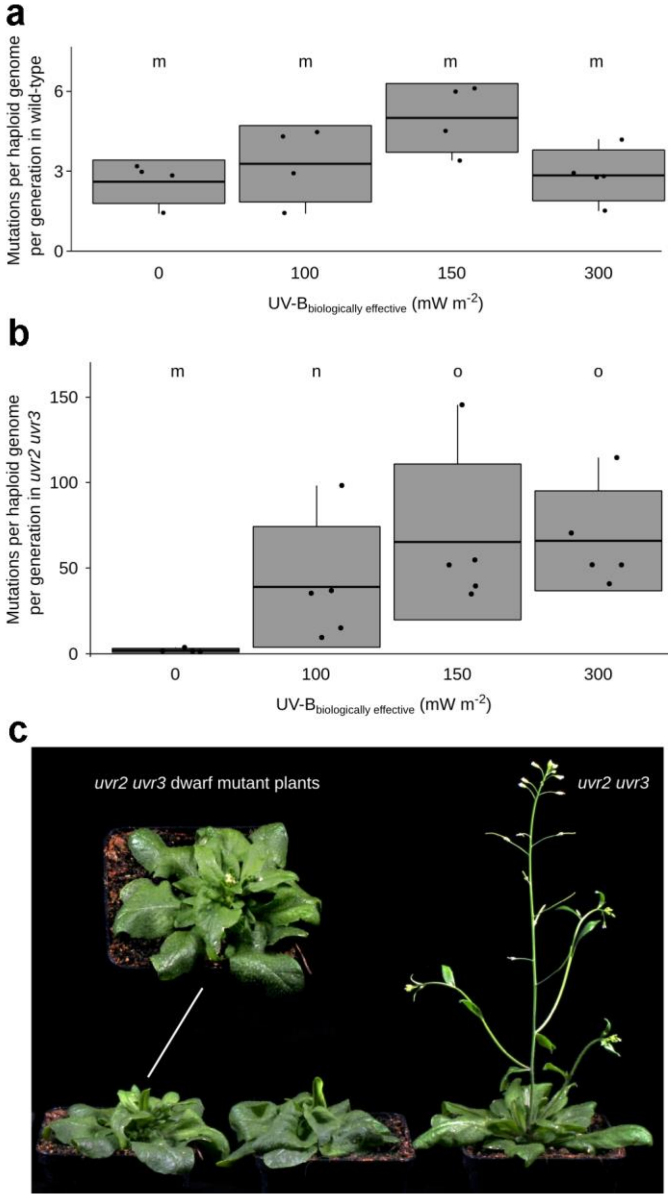

Supplementary Figure 3: Mutation accumulation under different UV-B conditions and generations.

(**a**) Number of mutations in wild-type plants after one generation at 0, 100, 150 and 300 mW m^{-2} biologically effective UV-B. m - None of the comparisons showed significant differences (Fisher's exact, *P = >0.05*). (**b**) Number of mutations in *uvr2 uvr3* plants under different UV-B radiation as described in (**c**). m – non-significant differences to control-treated wild-type (Fisher's exact, *P = >0.05*), n - significant differences compared to 0, 150 and 300 mW m^{-2} UV-B_{BE} (Fisher's exact, *P = <0.05*), o - significant differences compared to 0 and 100 mW m^{-2} UV-B_{BE} (Fisher's exact, *P = <0.05*). (**c**) Representative phenotype of semi-dominantly-inherited *uvr2 uvr3* dwarf mutant plants compared to *uvr2 uvr3* plant.

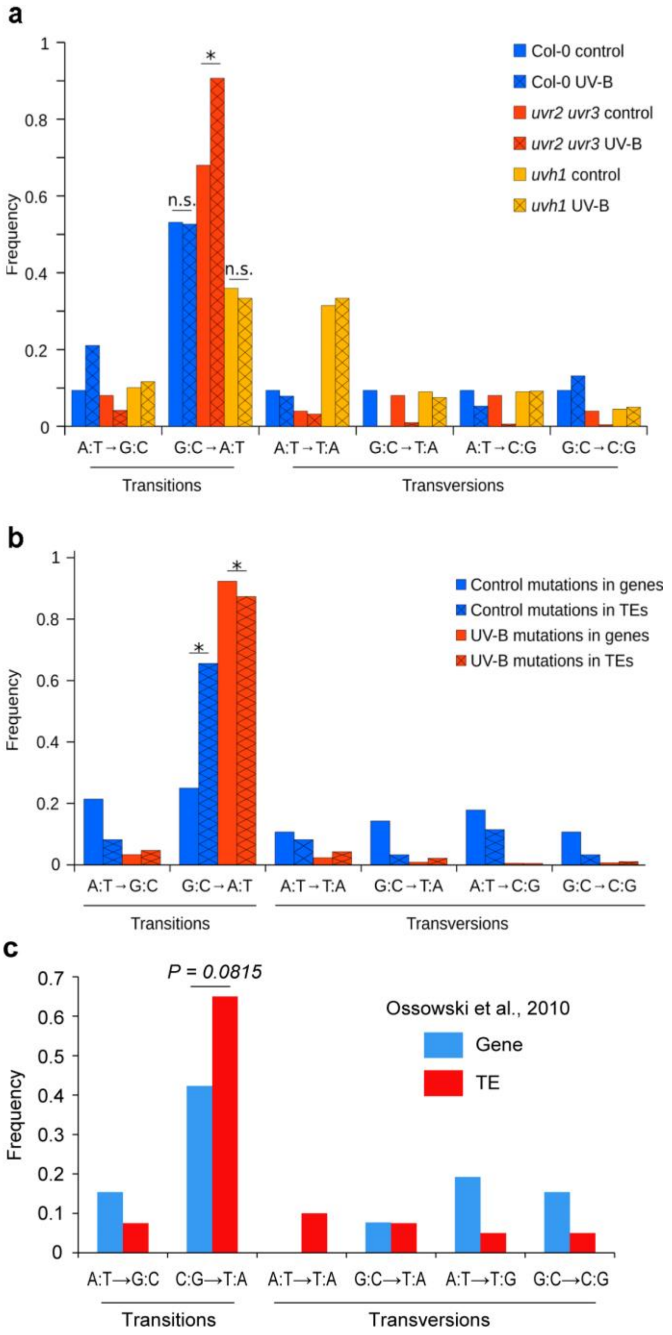

Supplementary Figure 4: Mutation spectra.

(**a**) Frequency of single nucleotide substitutions in control and UV-B treated Col-0 wild type, *uvr2 uvr3* and *uvh1* plants. The test results are shown only for G:C→A:T mutations representing the dominant group. * Fisher's exact test, $P < 0.05$. n.s. = not significant. (**b**) Frequency of single nucleotide changes in genes and transposable elements (TEs) collectively in all genotypes except for *uvh1*. Statistical evaluation was performed as described in (a). (**c**) Frequency of single nucleotide changes in genes and TEs based on data from Ossowski et al., 2010. Statistical evaluation was performed as described in (**a**).

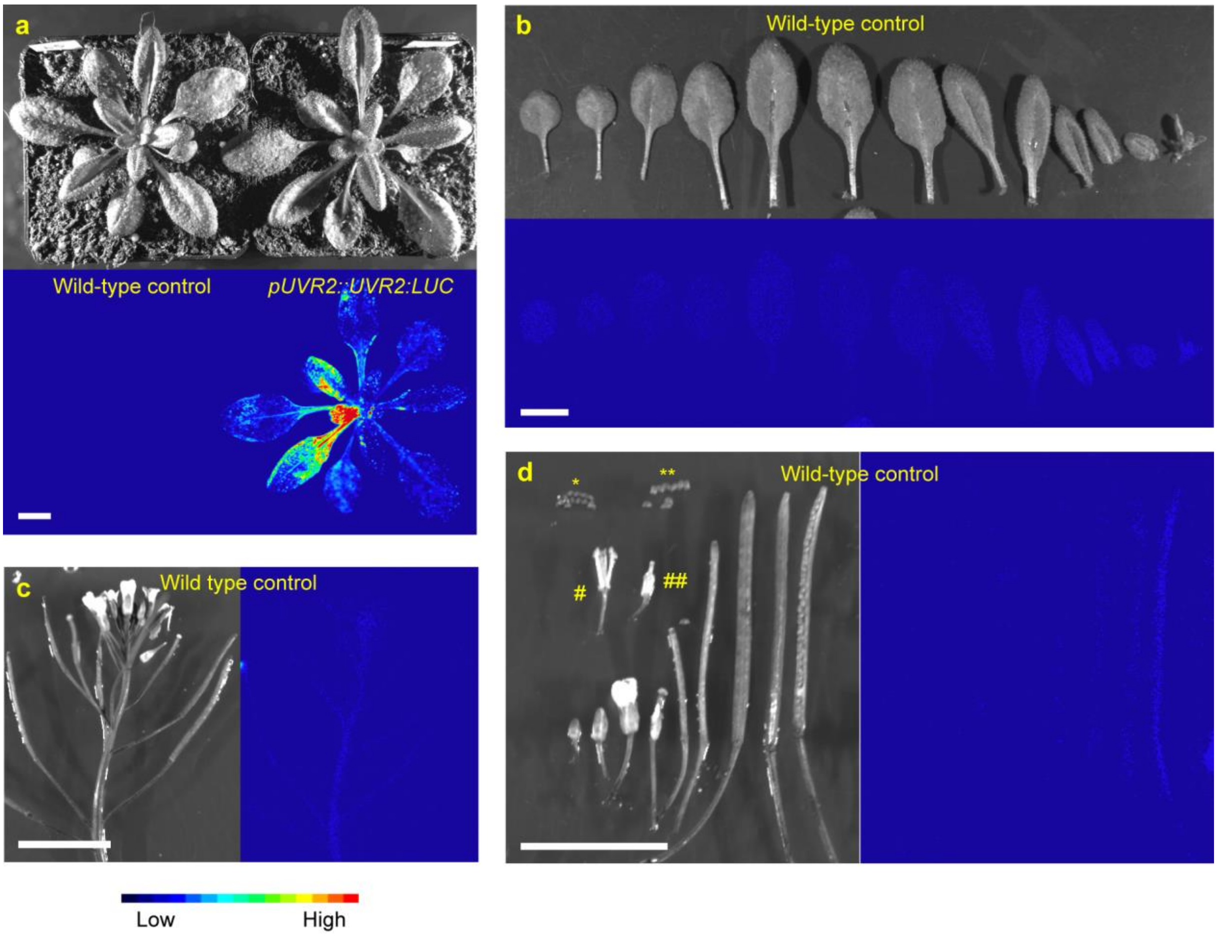

Supplementary Figure 5: UVR2 expression and background signals in control plants. Wild-type control plants without luciferase reporter construct and UVR2-LUCIFERASE translational fusion reporter line (*UVR2promoter::UVR2:LUCIFERASE*). Images on the top/left show plant tissues under white light and those on the bottom/right luciferase signal. All luciferase images were takes using identical exposure time of 1 min. Absence of signals in control plants should be compared with UVR2-LUCIFERASE signals in **Fig. 3b-e**. (**a**) Three weeks old wild-type control and UVR2-LUCIFERASE reporter line plants. (**b**) Leaves dissected from three weeks old control wild-type plant organized from the oldest (left) to the youngest (right). (**c**) Inflorescence of control wild-type. (**d**) Flower, silique and seed developmental series of control wild-type. Bottom row, left to right: closed flower, flower with emerging pistil, fully opened flower, siliques at different stages and the last opened silique containing seeds with mature embryos. Hashes: pistils and anthers from (#) opened and (##) closed flowers. Petals and sepals were manually removed. Asterisks: (*) dry and (**) fresh seeds. Bars = 10 mm. Color scale at the bottom indicates luciferase signal intensity.

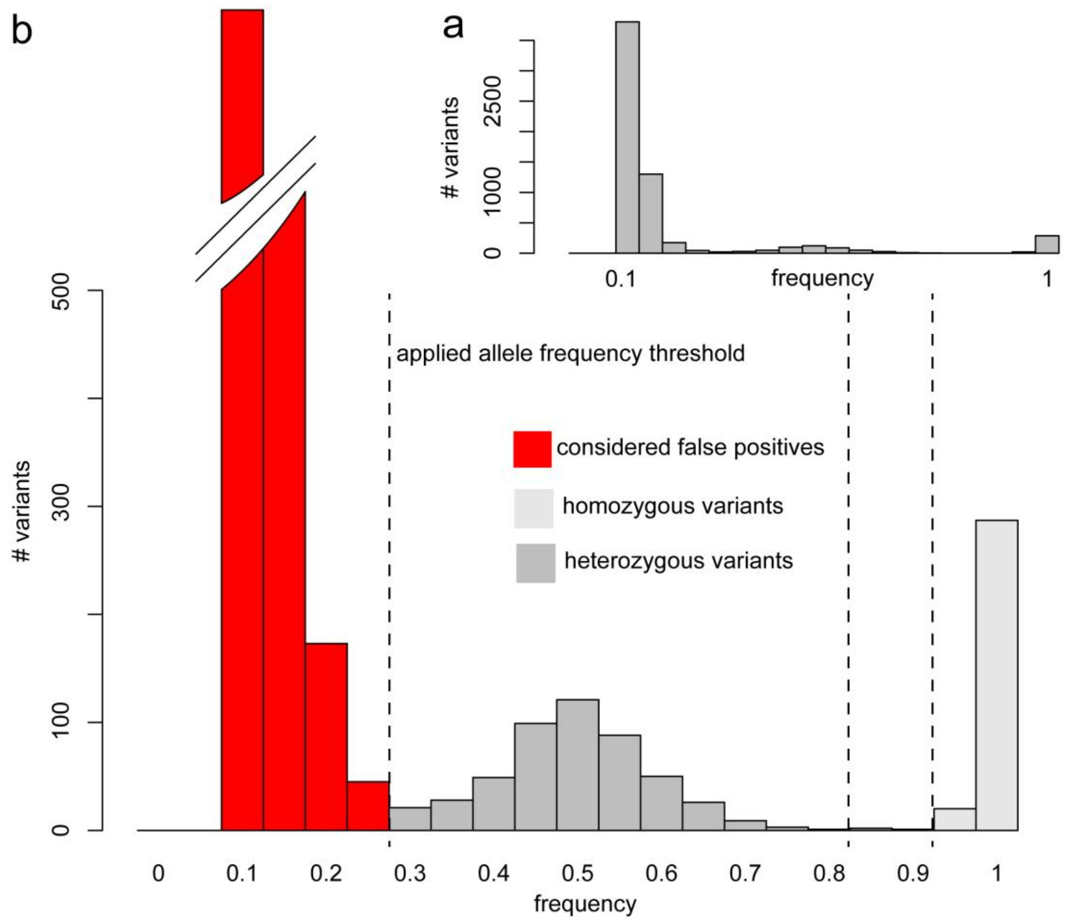

Supplementary Figure 6: **Allele frequency distribution of variable sites in a *uvr2 uvr3* genome.**

(**a**) Example of a frequency spectra of variable sites for a single *uvr2 uvr3* genome. After extracting genome specific variants as described in Material and Methods, allele frequency of more than 0.1 were assessed (excluding obvious sequencing errors). (**b**) Zoom in into (**a**). There is a clear cut between homozygous and heterozygous variants on the right half of the histogram. Assuming that heterozygous mutations display frequencies (0.1 <= x <= 0.9), which are normally distributed with a mean of 0.5, variants with a frequency < 0.3 seemingly include a lot of false positives. The minimal turning point at 0.3 serves as a cutoff to ensure that the majority of false positives are excluded from the analysis and only a very small number of true positives is lost.

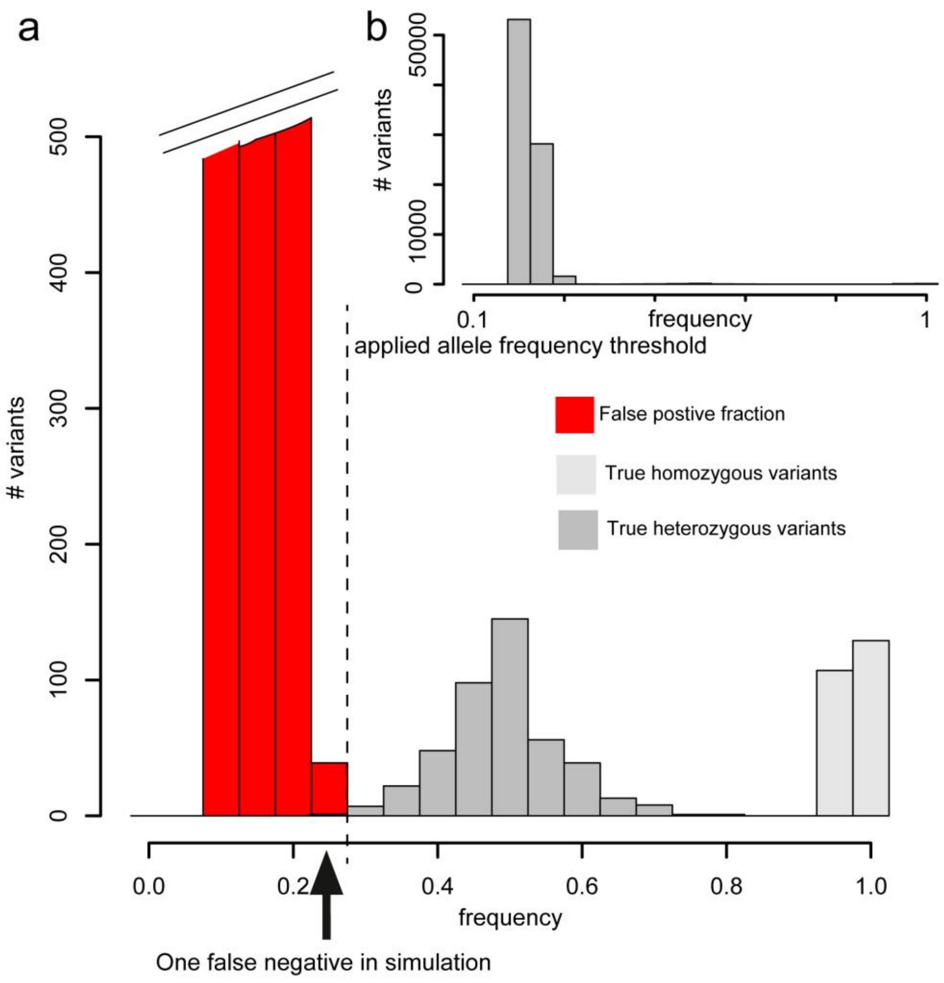

Supplementary Figure 7: **Allele frequency distribution of variable sites in simulated data.**

(**a**) Frequency spectra of variable sites specific to a simulated genome with 900 *in silico* mutations. (**b**) Zoom in into (**a**).

Supplementary Table 1: Mean number of mutations per genome in genic regions of control and UV-B-irradiated plants.

UVR2 included wild-type, *uvr8*, *tt4* and *uvr3* genotypes; *uvr2* included *uvr2* and *uvr2 uvr3* genotypes. *uvh1* was excluded from this analysis. n = number of analyzed genomes for individual groups.

	Control						UV-B					
	UVR2 (n = 29)			*uvr2* (n = 15)			*UVR2* (n = 37)			*uvr2* (n = 29)		
	Total	Per genome	%	Total	Per genome	%	Total	Per genome	%	Total	Per genome	%
Intergenic and TEs	63	2.2	74.1	31	2.1	67.4	112	3.0	70.0	1303	44.9	64.5
5' UTR	1	0.0	1.2	1	0.1	2.2	1	0.0	0.6	26	0.9	1.3
3' UTR	3	0.1	3.5	2	0.1	4.3	6	0.2	3.8	45	1.6	2.2
intron	10	0.3	11.8	4	0.3	8.7	15	0.4	9.4	250	8.6	12.4
CDS synonymous	3	0.1	3.5	2	0.1	4.3	7	0.2	4.4	98	3.4	4.9
CDS non-synonymous	5	0.2	5.9	6	0.4	13.0	19	0.5	11.9	297	10.2	14.7
**Sum**	**85**	**2.9**	100.0	**46**	**3.1**	100.0	**160**	**4.3**	100.0	**2019**	**69.6**	100.0

Supplementary Table 2: Sex-specific UV-B mutations.

F1 reciprocal hybrids of control and UV-B-irradiated *uvr2 uvr3* plants (see experimental design in Figure 3c) were analyzed in two independent biological replicates. Complete absence of homozygous mutations excluded contamination by self-pollination. The normalized frequencies of mutations were calculated using absolute numbers of heterozygous mutations and accessible genome space (Table S1).

Sex		Biological replicate	Genome replicate	Mutations per haploid genome and generation			Mean	Std. dev.
Female	Male			Homozygous	Heterozygous	Normalized*		
Control	UV-B-treated	1	1	0	11	13.6	13.3	9.0
		1	2	0	7	8.6		
		1	3	0	3	3.8		
		2	4	0	10	12.3		
		2	5	0	16	19.7		
		2	6	0	8	9.9		
		2	7	0	5	6.2		
		2	8	0	26	32.0		
UV-B-treated	Control	1	1	0	28	34.6	12.4	9.4
		1	2	0	8	9.9		
		1	3	0	7	8.6		
		2	4	0	4	4.9		
		2	5	0	10	12.3		
		2	6	0	5	6.2		
		2	7	0	7	8.6		
		2	8	0	11	13.7		

*Normalized to accepted positions per genome
